# Dibenzoatobis[3-(pyrrol-1-ylmeth­yl)pyridine]­zinc(II)

**DOI:** 10.1107/S1600536810028503

**Published:** 2010-07-21

**Authors:** Hong Gyu Lee, Jin Hoon Kim, Pan-Gi Kim, Cheal Kim, Youngmee Kim

**Affiliations:** a Department of Fine Chemistry and Eco-Product and Materials Education Center, Seoul National University of Technology, Seoul 139-743, Republic of Korea; b Department of Forest & Environment Resources, Kyungpook National University, Sangju 742-711, Republic of Korea; c Deaprtment of Chemistry and Nano Science, Ewha Womans University, Seoul 120-750, Republic of Korea

## Abstract

In the title compound, [Zn(C_7_H_5_O_2_)_2_(C_10_H_10_N_2_)_2_], the Zn^II^ ion, located on a twofold axis, is coordinated by two N atoms from two 3-(pyrrol-1-ylmeth­yl)pyridine ligands and two O atoms from two benzoate ligands in a distorted tetra­hedral geometry. The pyridine and the pyrrole rings are nearly perpendicular to each other, making a dihedral angle of 84.83 (7)°.

## Related literature

For examples of inter­actions between transition metal ions and biologically active mol­ecules, see: Daniele *et al.* (2008 ▶); Parkin (2004 ▶); Tshuva & Lippard (2004 ▶). For related structures, see: Lee *et al.* (2008 ▶); Park *et al.* (2008 ▶); Shin *et al.*(2009 ▶); Yu *et al.* (2008 ▶, 2009 ▶, 2010 ▶). For a description of the Cambridge Structural Database, see: Allen (2002 ▶).
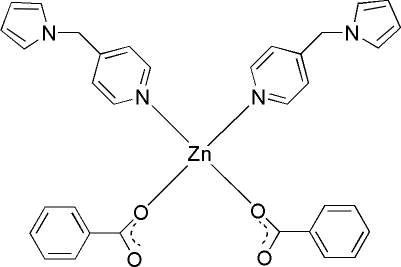


## Experimental

### 

#### Crystal data

[Zn(C_7_H_5_O_2_)_2_(C_10_H_10_N_2_)_2_]
*M* *_r_* = 623.99
Monoclinic, 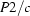

*a* = 14.4347 (14) Å
*b* = 9.4399 (9) Å
*c* = 11.1959 (11) Å
β = 102.896 (2)°
*V* = 1487.1 (2) Å^3^
*Z* = 2
Mo *K*α radiation
μ = 0.87 mm^−1^
*T* = 170 K
0.15 × 0.10 × 0.03 mm

#### Data collection

Bruker SMART CCD diffractometer
8090 measured reflections
2904 independent reflections
2204 reflections with *I* > 2σ(*I*)
*R*_int_ = 0.065

#### Refinement

*R*[*F* ^2^ > 2σ(*F* ^2^)] = 0.034
*wR*(*F* ^2^) = 0.071
*S* = 0.91
2904 reflections
195 parameters
H-atom parameters constrained
Δρ_max_ = 0.52 e Å^−3^
Δρ_min_ = −0.51 e Å^−3^

### 

Data collection: *SMART* (Bruker, 1997 ▶); cell refinement: *SAINT* (Bruker, 1997 ▶); data reduction: *SAINT*; program(s) used to solve structure: *SHELXS97* (Sheldrick, 2008 ▶); program(s) used to refine structure: *SHELXL97* (Sheldrick, 2008 ▶); molecular graphics: *ORTEPIII* (Burnett & Johnson, 1996 ▶) and *ORTEP-3 for Windows* (Farrugia, 1997 ▶); software used to prepare material for publication: *SHELXTL* (Sheldrick, 2008 ▶).

## Supplementary Material

Crystal structure: contains datablocks I, global. DOI: 10.1107/S1600536810028503/dn2587sup1.cif

Structure factors: contains datablocks I. DOI: 10.1107/S1600536810028503/dn2587Isup2.hkl

Additional supplementary materials:  crystallographic information; 3D view; checkCIF report

## Figures and Tables

**Fig. 1.**
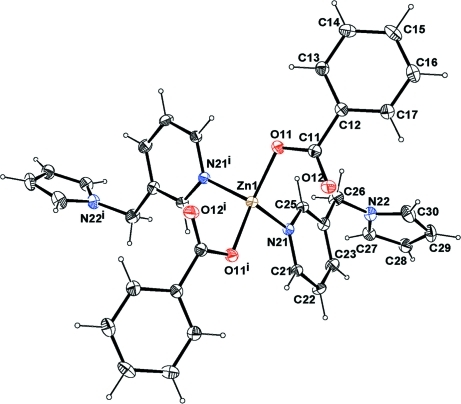
The structure of the title compound showing the atom-labeling scheme. Displacement ellipsoids are shown at the 30% probability level. H atoms are represented as samll spheres of arbitrary radii. [Symmetry code: (i) 1-x, y, 3/2-z].

## supplementary crystallographic information

### Comment

The interaction of transition metal ions with biologically active molecules such as amino acids, proteins, sugars, nucleotides *etc*, is of great importance in the study of biological systems (Daniele, *et al.*, 2008; Parkin, 2004; Tshuva & Lippard, 2004). As possible models for studying such interaction, the chemistry of transition metal ions with fulvic acids and humic acids has been intensively examined. Our group have reported a variety of structures of copper(II) and zinc(II) benzoates with quinoxaline, 6-methylquinoline, 3-methylquinoline, *trans*-1-(2-pyridyl)-2-(4-pyridyl)ethylene, and di-2-pyridyl ketone (Lee, *et al.*, 2008; Yu, *et al.*, 2008;Park, *et al.*, 2008; Shin, *et al.*,2009;Yu, *et al.*, 2009; Yu *et al.*,2010) in order to study the interaction of the transition metal ions with various acids. In this work, we have employed zinc(II) benzoate as a building block and 3-(pyrrol-1-ylmethyl)pyridine as a ligand.

In the title compound, the Zn^II^ ion is located on a two fold axis and is coordinated by two nitrogen atoms from two symmetry related 3-(pyrrol-1-ylmethyl)pyridine ligands and two oxygen atoms from two symmetry related benzoate ligands to form a distorted tetrahedral geometry (Fig. 1). Zn—N and Zn—O bond distances are in agreement with reported bond distances in the Cambridge Structural Database (Allen, 2002). The pyridine and the pyrrol rings are nearly perpendicular to each other making a dihedral angle of 84.83 (7)°.

### Experimental

30.4 mg (0.1 mmol) of Zn(NO_3_)_2_^.^6H_2_O and 28.0 mg (0.2 mmol) of C_6_H_5_COONH_4_ were dissolved in 4 ml H_2_O and carefully layered by 4 ml acetone solution of 3-(pyrrol-1-ylmethyl)pyridine (31.8 mg, 0.2 mmol). Suitable crystals of the title compound for X-ray analysis were obtained in a few weeks.

### Refinement

H atoms were placed in calculated positions and treated as riding with C—H distances of 0.95 Å (pyridine and pyrrolidine) and 0.99 Å (methylene) and with U_iso_(H)= 1.2U_eq_(C).

### Crystal data

**Table:**

[Zn(C_7_H_5_O_2_)_2_(C_10_H_10_N_2_)_2_]	*F*(000) = 648
*M**_r_* = 623.99	*D*_x_ = 1.394 Mg m^−^^3^
Monoclinic, *P*2/*c*	Mo *K*α radiation, λ = 0.71073 Å
Hall symbol: -P 2yc	Cell parameters from 2361 reflections
*a* = 14.4347 (14) Å	θ = 2.6–24.6°
*b* = 9.4399 (9) Å	µ = 0.87 mm^−^^1^
*c* = 11.1959 (11) Å	*T* = 170 K
β = 102.896 (2)°	Plate, colorless
*V* = 1487.1 (2) Å^3^	0.15 × 0.10 × 0.03 mm
*Z* = 2	

### Data collection

**Table:**

Bruker SMART CCD diffractometer	2204 reflections with *I* > 2σ(*I*)
Radiation source: fine-focus sealed tube	*R*_int_ = 0.065
graphite	θ_max_ = 26.0°, θ_min_ = 2.2°
φ and ω scans	*h* = −9→17
8090 measured reflections	*k* = −11→11
2904 independent reflections	*l* = −13→13

### Refinement

**Table:**

Refinement on *F*^2^	Primary atom site location: structure-invariant direct methods
Least-squares matrix: full	Secondary atom site location: difference Fourier map
*R*[*F*^2^ > 2σ(*F*^2^)] = 0.034	Hydrogen site location: inferred from neighbouring sites
*wR*(*F*^2^) = 0.071	H-atom parameters constrained
*S* = 0.91	*w* = 1/[σ^2^(*F*_o_^2^) + (0.0248*P*)^2^] where *P* = (*F*_o_^2^ + 2*F*_c_^2^)/3
2904 reflections	(Δ/σ)_max_ < 0.001
195 parameters	Δρ_max_ = 0.52 e Å^−^^3^
0 restraints	Δρ_min_ = −0.51 e Å^−^^3^

### Special details

**Table:**

Geometry. All e.s.d.'s (except the e.s.d. in the dihedral angle between two l.s. planes) are estimated using the full covariance matrix. The cell e.s.d.'s are taken into account individually in the estimation of e.s.d.'s in distances, angles and torsion angles; correlations between e.s.d.'s in cell parameters are only used when they are defined by crystal symmetry. An approximate (isotropic) treatment of cell e.s.d.'s is used for estimating e.s.d.'s involving l.s. planes.
Refinement. Refinement of *F*^2^ against ALL reflections. The weighted *R*-factor *wR* and goodness of fit *S* are based on *F*^2^, conventional *R*-factors *R* are based on *F*, with *F* set to zero for negative *F*^2^. The threshold expression of *F*^2^ > σ(*F*^2^) is used only for calculating *R*-factors(gt) *etc*. and is not relevant to the choice of reflections for refinement. *R*-factors based on *F*^2^ are statistically about twice as large as those based on *F*, and *R*- factors based on ALL data will be even larger.

### Fractional atomic coordinates and isotropic or equivalent isotropic displacement parameters (Å^2^)

**Table:**

	*x*	*y*	*z*	*U*_iso_*/*U*_eq_	
Zn1	0.5000	0.58373 (4)	0.7500	0.02372 (12)	
O11	0.39278 (9)	0.51333 (15)	0.63040 (12)	0.0289 (4)	
O12	0.36133 (10)	0.38352 (16)	0.78176 (13)	0.0376 (4)	
C11	0.33872 (14)	0.4306 (2)	0.67596 (19)	0.0259 (5)	
C12	0.24503 (14)	0.3958 (2)	0.59353 (18)	0.0241 (5)	
C13	0.22904 (14)	0.4186 (2)	0.46787 (18)	0.0294 (5)	
H13	0.2794	0.4511	0.4330	0.035*	
C14	0.14056 (16)	0.3944 (2)	0.3935 (2)	0.0364 (6)	
H14	0.1303	0.4094	0.3076	0.044*	
C15	0.06679 (16)	0.3482 (2)	0.4441 (2)	0.0395 (6)	
H15	0.0056	0.3330	0.3930	0.047*	
C16	0.08190 (16)	0.3240 (3)	0.5688 (2)	0.0387 (6)	
H16	0.0311	0.2924	0.6034	0.046*	
C17	0.17063 (15)	0.3458 (2)	0.6428 (2)	0.0310 (5)	
H17	0.1813	0.3266	0.7281	0.037*	
N21	0.45103 (11)	0.72476 (17)	0.86239 (14)	0.0238 (4)	
N22	0.22689 (12)	1.08801 (19)	0.91482 (16)	0.0316 (4)	
C21	0.48598 (14)	0.7216 (2)	0.98335 (18)	0.0258 (5)	
H21	0.5324	0.6523	1.0162	0.031*	
C22	0.45709 (15)	0.8149 (2)	1.06183 (19)	0.0325 (5)	
H22	0.4824	0.8088	1.1476	0.039*	
C23	0.39112 (15)	0.9174 (2)	1.01497 (19)	0.0328 (5)	
H23	0.3708	0.9829	1.0682	0.039*	
C24	0.35458 (14)	0.9243 (2)	0.88950 (19)	0.0283 (5)	
C25	0.38588 (14)	0.8249 (2)	0.81762 (18)	0.0258 (5)	
H25	0.3602	0.8270	0.7318	0.031*	
C26	0.28375 (16)	1.0365 (2)	0.8315 (2)	0.0380 (6)	
H26A	0.3184	1.1171	0.8053	0.046*	
H26B	0.2410	0.9966	0.7576	0.046*	
C27	0.24005 (15)	1.2131 (2)	0.9763 (2)	0.0337 (5)	
H27	0.2843	1.2847	0.9675	0.040*	
C28	0.17895 (16)	1.2179 (3)	1.0527 (2)	0.0389 (6)	
H28	0.1725	1.2932	1.1065	0.047*	
C29	0.12722 (16)	1.0912 (3)	1.0374 (2)	0.0443 (6)	
H29	0.0790	1.0649	1.0786	0.053*	
C30	0.15875 (16)	1.0129 (3)	0.9527 (2)	0.0429 (6)	
H30	0.1368	0.9212	0.9248	0.051*	

### Atomic displacement parameters (Å^2^)

**Table:**

	*U*^11^	*U*^22^	*U*^33^	*U*^12^	*U*^13^	*U*^23^
Zn1	0.0232 (2)	0.0247 (2)	0.0228 (2)	0.000	0.00434 (14)	0.000
O11	0.0247 (8)	0.0311 (9)	0.0300 (8)	−0.0069 (7)	0.0045 (7)	−0.0004 (7)
O12	0.0319 (9)	0.0500 (11)	0.0286 (9)	−0.0016 (7)	0.0017 (7)	0.0079 (7)
C11	0.0262 (12)	0.0236 (12)	0.0279 (12)	0.0047 (10)	0.0060 (9)	−0.0044 (10)
C12	0.0244 (11)	0.0186 (11)	0.0291 (12)	0.0026 (9)	0.0053 (9)	−0.0004 (9)
C13	0.0300 (12)	0.0264 (12)	0.0319 (12)	−0.0026 (10)	0.0073 (10)	−0.0006 (10)
C14	0.0398 (14)	0.0333 (14)	0.0307 (13)	−0.0035 (11)	−0.0039 (11)	0.0008 (10)
C15	0.0244 (13)	0.0347 (14)	0.0524 (16)	−0.0013 (11)	−0.0066 (11)	−0.0031 (12)
C16	0.0274 (13)	0.0373 (14)	0.0528 (16)	−0.0030 (11)	0.0121 (12)	−0.0014 (12)
C17	0.0303 (13)	0.0293 (12)	0.0337 (13)	0.0007 (10)	0.0078 (10)	0.0015 (10)
N21	0.0230 (10)	0.0257 (10)	0.0228 (9)	−0.0016 (7)	0.0054 (7)	0.0014 (8)
N22	0.0309 (10)	0.0248 (10)	0.0403 (11)	0.0040 (9)	0.0104 (8)	0.0000 (9)
C21	0.0243 (12)	0.0289 (12)	0.0240 (12)	0.0007 (9)	0.0051 (9)	0.0047 (10)
C22	0.0374 (14)	0.0400 (14)	0.0196 (11)	−0.0015 (11)	0.0052 (10)	0.0004 (10)
C23	0.0416 (14)	0.0297 (12)	0.0290 (12)	0.0012 (11)	0.0121 (10)	−0.0053 (11)
C24	0.0312 (12)	0.0252 (12)	0.0296 (12)	0.0010 (10)	0.0094 (10)	0.0008 (10)
C25	0.0266 (12)	0.0290 (12)	0.0209 (11)	0.0006 (10)	0.0038 (9)	0.0017 (10)
C26	0.0456 (15)	0.0331 (14)	0.0367 (14)	0.0114 (11)	0.0122 (12)	0.0002 (11)
C27	0.0319 (13)	0.0268 (13)	0.0417 (14)	0.0008 (10)	0.0070 (11)	−0.0017 (11)
C28	0.0350 (15)	0.0418 (15)	0.0410 (14)	0.0066 (11)	0.0107 (12)	−0.0063 (12)
C29	0.0298 (13)	0.0471 (16)	0.0604 (17)	0.0038 (12)	0.0197 (12)	0.0083 (14)
C30	0.0353 (15)	0.0275 (14)	0.0649 (18)	−0.0029 (11)	0.0093 (13)	−0.0004 (12)

### Geometric parameters (Å, °)

**Table:**

Zn1—O11^i^	1.9248 (13)	N22—C27	1.359 (3)
Zn1—O11	1.9248 (13)	N22—C26	1.457 (3)
Zn1—N21^i^	2.0621 (16)	C21—C22	1.373 (3)
Zn1—N21	2.0621 (16)	C21—H21	0.9500
O11—C11	1.287 (2)	C22—C23	1.377 (3)
O12—C11	1.239 (2)	C22—H22	0.9500
C11—C12	1.494 (3)	C23—C24	1.387 (3)
C12—C13	1.391 (3)	C23—H23	0.9500
C12—C17	1.395 (3)	C24—C25	1.376 (3)
C13—C14	1.379 (3)	C24—C26	1.514 (3)
C13—H13	0.9500	C25—H25	0.9500
C14—C15	1.383 (3)	C26—H26A	0.9900
C14—H14	0.9500	C26—H26B	0.9900
C15—C16	1.384 (3)	C27—C28	1.359 (3)
C15—H15	0.9500	C27—H27	0.9500
C16—C17	1.377 (3)	C28—C29	1.400 (3)
C16—H16	0.9500	C28—H28	0.9500
C17—H17	0.9500	C29—C30	1.359 (3)
N21—C21	1.336 (2)	C29—H29	0.9500
N21—C25	1.349 (2)	C30—H30	0.9500
N22—C30	1.355 (3)		
			
O11^i^—Zn1—O11	139.60 (9)	N21—C21—C22	122.29 (19)
O11^i^—Zn1—N21^i^	108.40 (6)	N21—C21—H21	118.9
O11—Zn1—N21^i^	97.49 (6)	C22—C21—H21	118.9
O11^i^—Zn1—N21	97.48 (6)	C21—C22—C23	119.3 (2)
O11—Zn1—N21	108.39 (6)	C21—C22—H22	120.3
N21^i^—Zn1—N21	99.58 (9)	C23—C22—H22	120.3
C11—O11—Zn1	113.47 (13)	C22—C23—C24	119.6 (2)
O12—C11—O11	122.87 (19)	C22—C23—H23	120.2
O12—C11—C12	121.42 (19)	C24—C23—H23	120.2
O11—C11—C12	115.70 (18)	C25—C24—C23	117.37 (19)
C13—C12—C17	118.95 (19)	C25—C24—C26	120.30 (18)
C13—C12—C11	120.95 (18)	C23—C24—C26	122.32 (19)
C17—C12—C11	120.04 (18)	N21—C25—C24	123.58 (18)
C14—C13—C12	120.5 (2)	N21—C25—H25	118.2
C14—C13—H13	119.8	C24—C25—H25	118.2
C12—C13—H13	119.8	N22—C26—C24	112.46 (17)
C13—C14—C15	119.9 (2)	N22—C26—H26A	109.1
C13—C14—H14	120.0	C24—C26—H26A	109.1
C15—C14—H14	120.0	N22—C26—H26B	109.1
C14—C15—C16	120.2 (2)	C24—C26—H26B	109.1
C14—C15—H15	119.9	H26A—C26—H26B	107.8
C16—C15—H15	119.9	N22—C27—C28	108.1 (2)
C17—C16—C15	119.9 (2)	N22—C27—H27	125.9
C17—C16—H16	120.0	C28—C27—H27	125.9
C15—C16—H16	120.0	C27—C28—C29	107.3 (2)
C16—C17—C12	120.5 (2)	C27—C28—H28	126.3
C16—C17—H17	119.8	C29—C28—H28	126.3
C12—C17—H17	119.8	C30—C29—C28	107.3 (2)
C21—N21—C25	117.83 (17)	C30—C29—H29	126.3
C21—N21—Zn1	120.05 (14)	C28—C29—H29	126.3
C25—N21—Zn1	122.09 (13)	N22—C30—C29	108.3 (2)
C30—N22—C27	108.97 (18)	N22—C30—H30	125.9
C30—N22—C26	125.30 (19)	C29—C30—H30	125.9
C27—N22—C26	125.43 (19)		
			
O11^i^—Zn1—O11—C11	56.95 (13)	N21^i^—Zn1—N21—C25	56.41 (14)
N21^i^—Zn1—O11—C11	−172.84 (13)	C25—N21—C21—C22	0.7 (3)
N21—Zn1—O11—C11	−70.10 (14)	Zn1—N21—C21—C22	178.71 (15)
Zn1—O11—C11—O12	−10.7 (3)	N21—C21—C22—C23	−1.2 (3)
Zn1—O11—C11—C12	168.33 (12)	C21—C22—C23—C24	0.3 (3)
O12—C11—C12—C13	−164.0 (2)	C22—C23—C24—C25	1.0 (3)
O11—C11—C12—C13	17.0 (3)	C22—C23—C24—C26	−178.4 (2)
O12—C11—C12—C17	18.9 (3)	C21—N21—C25—C24	0.8 (3)
O11—C11—C12—C17	−160.13 (18)	Zn1—N21—C25—C24	−177.24 (15)
C17—C12—C13—C14	1.1 (3)	C23—C24—C25—N21	−1.6 (3)
C11—C12—C13—C14	−176.02 (19)	C26—C24—C25—N21	177.83 (19)
C12—C13—C14—C15	0.5 (3)	C30—N22—C26—C24	−70.4 (3)
C13—C14—C15—C16	−1.1 (3)	C27—N22—C26—C24	102.6 (2)
C14—C15—C16—C17	−0.1 (4)	C25—C24—C26—N22	153.59 (19)
C15—C16—C17—C12	1.8 (3)	C23—C24—C26—N22	−27.0 (3)
C13—C12—C17—C16	−2.3 (3)	C30—N22—C27—C28	−0.7 (3)
C11—C12—C17—C16	174.9 (2)	C26—N22—C27—C28	−174.69 (19)
O11^i^—Zn1—N21—C21	−11.37 (15)	N22—C27—C28—C29	0.2 (3)
O11—Zn1—N21—C21	137.18 (14)	C27—C28—C29—C30	0.3 (3)
N21^i^—Zn1—N21—C21	−121.55 (16)	C27—N22—C30—C29	0.9 (3)
O11^i^—Zn1—N21—C25	166.58 (15)	C26—N22—C30—C29	174.90 (19)
O11—Zn1—N21—C25	−44.86 (16)	C28—C29—C30—N22	−0.8 (3)

Symmetry codes: (i) −*x*+1, *y*, −*z*+3/2.
